# Analysis of the omega-3 fatty acid content of South African fish oil supplements: a follow-up study

**DOI:** 10.5830/CVJA-2013-074

**Published:** 2013-10

**Authors:** Maretha Opperman, Spinnler Benade

**Affiliations:** Functional Foods Research Unit, Department of Agriculture and Food Science, Cape Peninsula University of Technology, Cape Town, South Africa; Functional Foods Research Unit, Department of Agriculture and Food Science, Cape Peninsula University of Technology, Cape Town, South Africa

**Keywords:** omega-3 fatty acids, supplement labels, conjugated dienes, peroxides, ethyl esters

## Abstract

**Introduction:**

Globally the omega-3 (n-3) fatty acid supplement industry is expanding rapidly while consumers are becoming more aware of the health benefits of n-3 fatty acids. Our group conducted a survey in 2009 on 45 commercially available fish oil supplements on the South African market. The aim of the study was to determine the fatty acid composition and content of supplements for comparison with the claimed contents on the product label. The survey was repeated in 2012 on 63 supplements.

**Methods:**

Sixty-three commercially available n-3 fatty acid supplements were analysed using gas–liquid chromatography to determine their fatty acid composition and content.

**Results:**

This analysis has shown an improvement in the accuracy of EPA content (44% in 2009) declared on supplement labels compared to the 2012 (52%) survey. It was also evident that a higher percentage of supplements (13% in 2009 vs 35% in 2012) contained DHA levels higher than declared. In 2009, 64% of supplements cost R2.01 to R5.00 or more to achieve a daily intake of 500 mg EPA + DHA, compared to 81% in 2012. Forty-four per cent of supplements were found to be in the early stages of rancidity [conjugated diene (CD) levels] compared to 73% in 2009. More than 80% of supplements had peroxide levels higher than the recommended content as specified by the Global Organisation for EPA and DHA Omega-3 (GOED). The majority (81%; *n* = 51) of the supplements under study in 2012 had a 1.1–1.5:1 EPA-to-DHA ratio or less, compared to 56% in 2009. Almost a third (32%) of the supplements in the 2012 survey contained ethyl esters (EE) or a combination of ethyl esters and triglycerides.

**Conclusion:**

Although the results of the 2012 versus the 2009 analysis were encouraging in terms of the accuracy of EPA declared on the supplement labels, the high peroxide levels found in the supplement oils are of concern. High peroxide levels are associated with potential health implications. EE were present in some of the supplements, even though the safety of EE has not been confirmed in vulnerable groups such as pregnant women and children.

Scientific research and media exposure as well as raising health and nutritional awareness among consumers have led to a substantial rise in fish oil supplementation over the past five years. It is predicted that sales of omega-3 (n-3) fatty acid products in the United States (US) will increase from $25.4 billion in 2011 to $34.7 billion in 2016 (pers commun).

Even though n-3 fatty acid supplements capture only 13% of the US n-3 product market, the highest retail dollar value of the supplements is in the fish oil as opposed to packaged food, beverages and infant formulas where the n-3 content is relatively small. The South African market for fish oil capsules over the past 12 months is estimated to be worth about R65 million (pers commun), however this figure only includes data from large retail store groups and not from privately owned businesses.

A whole array of health benefits are attributed to the long-chain polyunsaturated n-3 fatty acids found in fish oil. The n-3 fatty acids of particular interest are eicosapentaenoic acid (EPA) and docosahexaenoic acid (DHA). These fatty acids are eminent as anti-thrombotic, anti-arrhythmic and anti-aggregatory compounds.^1^

Routine intake of oily fish and fish oil are now widely acknowledged for their role in decreasing the risk of cardiovascular diseases (CVD) such as fatal coronary heart disease and stroke. N-3 fatty acids are also renowned for their anti-inflammatory properties and may be beneficial in reducing the risk of inflammatory-related conditions including obesity, Crohn’s disease, ulcerative colitis, type 2 diabetes, asthma, psoriasis, multiple sclerosis, cystic fibrosis and chronic obstructive pulmonary disease.^2^ The significance of EPA and DHA in neural and visual development is also well documented.^3^

In a typical Western diet there are only a few food sources contributing to dietary n-3 fatty acid intake. Research indicates that plant-derived n-3 fatty acids in the form of alpha-linolenic acid (ALA) are poorly converted to EPA and DHA,^4^ while preformed EPA and DHA from fish oil is readily available for metabolism. Several clinical trials have shown that baseline EPA and DHA blood levels can be increased by means of good-quality fish oil supplements.

Clinical trial results from our group indicated low dietary EPA and DHA intakes, as reflected in the baseline blood levels of healthy, normo-lipidaemic, non-smoking research participants residing in Cape Town. Typical baseline red blood cell values varied between 3 and 5%.^5^ According to Harris’s proposed n-3 index,^6^ these levels position participants in a high- to intermediate- risk zone for the development of CVD. As Cape Town is located on the west coast of South Africa with its cold sea currents, one could expect that cold-water fatty fish consumption will be higher among people residing here compared to residents living in the provinces without a coastline. Consumers further away from the coast may therefore experience even lower n-3 fatty acid levels in their blood.

However, canned fatty fish such as pilchards, sardines, salmon and mackerel are rich in n-3 fatty acids. These products are readily available to the majority of consumers and should theoretically help them to obtain their daily n-3 fatty acid intakes. Unfortunately the affordability of the products as well as some consumers’ preferences for red meat as a protein source may prevent them from purchasing these products. It therefore seems that progressively more consumers turn to fish oil supplements to augment their n-3 fatty acid intakes, as it is perceived to be an easy, effective and safe method to rectify poor dietary intakes.

Yet controversy exists whether n-3 fatty acids consumed via supplementation is the best way to combat disease. Some reviews investigating the risk of major cardiovascular events reported no association between n-3 fatty acid supplementation and a reduced risk of all-cause mortality, cardiac death, sudden death, myocardial infarction or stroke,^7^ or for the secondary prevention of cardiovascular disease.^8^ On the other hand, other reviews^9^,^10^ did indicate associations between risk reduction of cardiovascular disease and n-3 fatty acid dietary supplements. According to Jump *et al*.,^9^ conflicting results may be attributed to people at risk for cardiovascular disease who take medications such as statins and fibrates as well as drugs with anti-inflammatory, anti-arrhythmic and anti-thrombotic effects, minimising the detectable effect of supplemental n-3 fatty acids.

Dietary recommendations and advice for the intake of n-3 fatty acids have progressed notably during the past five years. No formal dietary reference intakes (DRIs) currently exist for EPA and DHA but intakes that vary from 250 to 600 mg EPA + DHA are recommended; however there are still many gaps in research^7^.

Achieving these amounts through fish intake alone might be problematic without supplementation. To reach a 500-mg/day EPA + DHA as recommended by the International Society for Fatty Acids and Lipids (ISSFAL^12^) with purified fish oil, about 2 000 mg of oil with a 180-mg EPA to 120-mg DHA content ratio would be required. To consume these amounts of EPA + DHA through diet/non-supplementation, approximately 79 g/day of tuna or 60 g/day of pilchards or 52 g/day of salmon should be consumed. In addition, fish is not the dietary protein of choice for many South Africans.

For an n-3 fatty acid supplementation to be effective, good-quality fish oil is essential. The quality of fish oil supplements is highly affected by the storage conditions of the refined oil before encapsulation, the nature of the preservative added to prevent oxidation, the time period of storage and light exposure. The EPA and DHA content of fish oil is also subjective to the fluctuation of long chain n-3 fatty acids even within single fish species and is further dependent on geographic origin, season and preparation of the oil.

In a survey^13^ conducted in 2009 by our research group, where we studied the quality of 45 fish oil supplements available on the South African market, it appeared that supplements varied to a large extent in terms of the measured versus the claimed content of EPA and/or DHA, the level of lipid peroxidation, the EPA-to-DHA ratios as well as the number of capsules and price to meet international dietary recommendations. These findings are of concern since no regulatory structure to monitor the quality of dietary supplements currently exists in South Africa. Consumers therefore depend on self-regulation within the neutraceutical industry for assurance of product quality, consistency, potency and purity of fish oil supplements.

Since the survey was conducted in 2009, no new labelling and quality-assurance regulations have been published. It was therefore decided to repeat the survey, this time on 63 supplements, to assess if there were any improvements in the quality of fish oil supplements available to the South African consumer. Specific objectives for the survey included analysis and comparison of the EPA and DHA contents of the supplements with the contents claimed on the product label; number of capsules and price to achieve international n-3 fatty acid intake recommendations; to determine the conjugated diene (CD) and peroxide content of the supplement oils; and to determine whether the long-chain n-3 fatty acids present in the capsules were in the form of triglycerides (TG) or ethyl esters (EE) or a combination of TG and EE.

## Methods

Sixty-three n-3 fatty acid supplements were analysed for fatty acid composition and content, CD and peroxide values as well as EE content. Two independent researchers de-identified the supplements and stored them in numbered containers until commencement of analyses, hence a blind sample of supplements was received by the researchers conducting the analyses. Products from various brands were analysed, and in no particular order included: Pharmamark, ReVite, ADD Vance, Bettaway, Vitaforce, Biogen, Amipro Metagenics, Keynote Health, Dis-Chem, Holistix, Naturelle, Natrodale, SOLAL Technologies, Medi+Rite Pharmacy, Nativa, Pharmafrica, Clicks Group Limited, Georen Preg, Bioharmony, Rejuvenesse, Solgar, Amway, Pinnacle, Scientific Sports Nutrition, Equazen, Patrick Holford, The Real Thing, Durbell, Vital, Holgoun Healthcare, Absolute Organix, Vitabiotics, Kenzahealth, Annique, Bioter, Ocean Gold Fish Liver Oil, and TNP Healthcare.

To determine the CD content of the fish oil supplements, a spectrophotometric method was used as described by Recknagel and Glende.^14^ CD values were compared against reference values as described by Opperman *et al.*^13^ Peroxide levels were determined spectrophotometrically as described by Khodaparast *et al.*^15^

## Fatty acid composition and content of fish oil capsules

The oil content of each capsule was determined by the difference in weight between the whole capsule and the weight of the capsule gelatin. An aliquot of oil, about 600 mg, was weighed in a 25-ml volumetric flask and made up to volume with chloroform–methanol (2:1) containing butylated hydroxyl toluene as anti-oxidant. To 50 μl of this stock solution was added 100 μl of heptadecanoic acid as internal standard (100 μl in chloroform-methanol 2:1).

After drying under a stream of nitrogen, fatty acids were trans-methylated with methanol-sulphuric acid (5% sulphuric acid in anhydrous methanol) for two hours at 70°C. Fatty acid methyl esters were extracted with hexane and analysed by gas–liquid chromatography (GLC, Thermo, Focus) equipped with a flame ionisation detector and a 60-meter BPX 70 capillary column. Oven temperature was programmed at 2°C per minute from 160 to 220°C.

Fatty acids were quantified by comparing the areas of a specific fatty acid with that of the internal standard and correcting for the relative response factor for the specific acid. Total fatty acids were calculated and each fatty acid was expressed as a percentage of the total fatty acids. The values so obtained were then translated into the amount of fatty acid per capsule.

## Determination of triglyceride and ethyl ester contents

The content of all fish oil capsules were screened for neutral lipid and EE content by high-performance thin-layer chromatography (HPTLC) using 10 × 10-cm silica gel 60 plates (Merck). Plates were developed with hexane–diethyl ether–acetic acid (85:15:2 by volume).^16^ Ten micro-samples (10 μl), prepared for GLC analysis of fatty acid, were applied to plates as a single spot.

Samples of EE of fatty acids and fatty acid TG were applied for identification. Lipids were identified by spraying with 2,5-Bis (5-tert-butyl-benzoxazol-2-yl) thiophene and visualised under UV light. Individual spots were scraped off the plate and eluted with chloroform methanol. TG spots were transmethylated with methanol sulphuric acid, where after EE spots were confirmed by GC-MS analysis.

## Results

Measured EPA and DHA contents of fish oil supplements were compared to the contents claimed on the product labels. A reference range between 90 and 110% of the manufacturers’ claimed contents for EPA and DHA was proposed. Supplements containing ≤ 89% of the claimed EPA and/or DHA were considered substandard, while those containing ≥ 110% EPA and/or DHA were considered to be in surplus.

CD contents of the fish oils were compared to levels as described by Opperman *et al.*^13^ For peroxide contents, values ≤ 5 meq O_2_/kg oil were deemed acceptable as recommended by the Global Organisation for EPA and DHA Omega-3 (GOED).^17^ Results of the 2012 study were compared to the results of a similar study conducted in 2009 to determine if there was any improvement in the accuracy of labelling information, the level of rancidity, and the EPA-to-DHA ratios of the supplements.

**Fig. 1.**
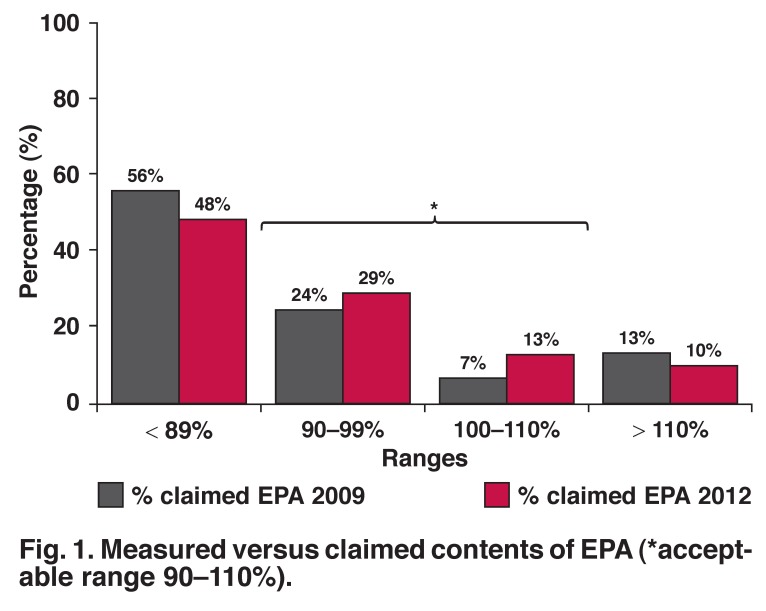
Measured versus claimed contents of EPA (*acceptable range 90–110%).

Comparison of measured versus claimed contents against the acceptable ranges (Fig. 1) indicated that almost half (*n* = 30; 48%) of the studied supplements failed to meet the claimed EPA requirements (2012). This is a small improvement compared to the 2009 analysis.

**Fig. 2.**
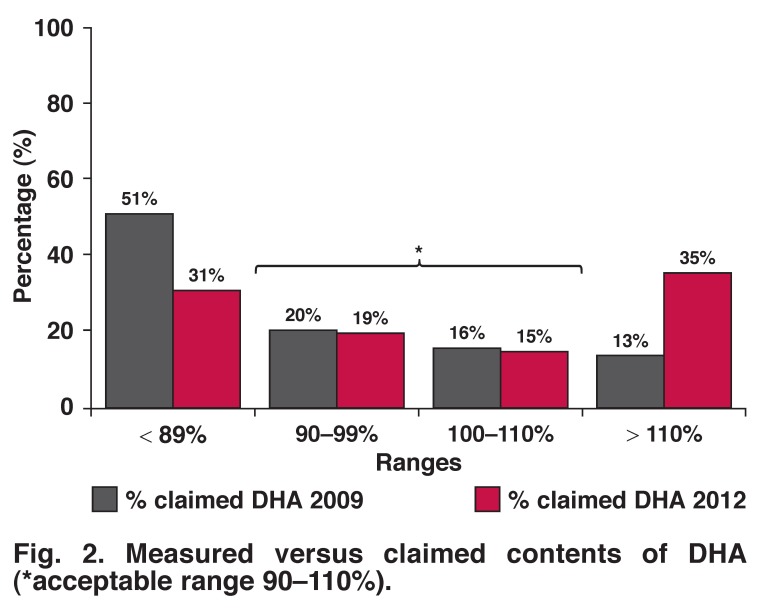
Measured versus claimed contents of DHA (*acceptable range 90–110%).

For DHA, 31% (*n* = 19) of the supplements did not meet the proposed DHA requirements (Fig. 2) compared to the 51% in the 2009 study. Thirty-five per cent (*n* = 22) of the supplements contained more than 110% of the DHA claimed on the product label. For the current EPA and DHA analysis, data of only 62 supplements could be recorded because one of the supplements did not provide any information on the label about the EPA and DHA content of the product.

**Fig. 3.**
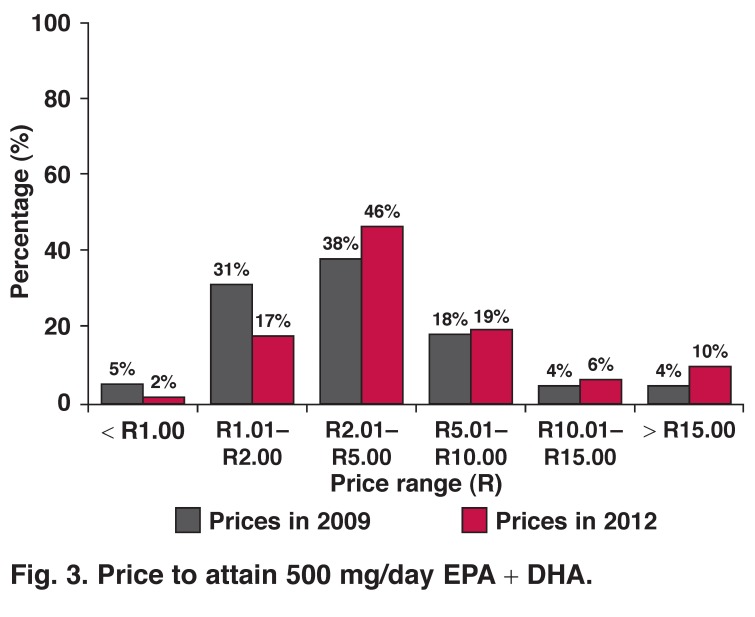
Price to attain 500 mg/day EPA + DHA.

In the absence of n-3 fatty acid dietary recommendations for South Africa, we used the ISSFAL^18^ recommended intake of 500 mg/day EPA + DHA for the prevention of cardiovascular disease, as reference. The majority (46%; *n* = 29) of the surveyed supplements cost between R2.01 and R5.00 to attain a 500-mg/day EPA + DHA intake. Thirty-five per cent (*n* = 22) of the supplements cost more than R5.00 per day to reach the proposed n-3 fatty acid intake (Fig. 3). Prices between R26.79 and R61.77 to achieve the daily recommended intake were recorded. Price increases seem to have been substantial between 2009 and 2012.

**Fig. 4.**
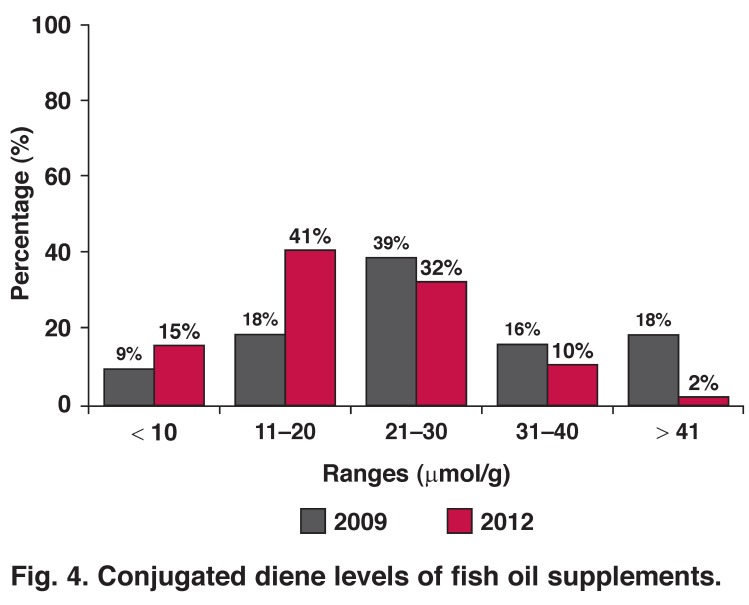
Conjugated diene levels of fish oil supplements.

The CD levels of oils are an indication of the early stages of rancidity of oils. CD levels of ≤ 20 μmol/g were considered acceptable. During the 2012 study, 56% (*n* = 33) of the analysed fish oil supplements contained CD levels within the acceptable ranges, while 44% (*n* = 26) had CD levels of > 20 μmol/g (Fig. 4).

CD levels of only 59 of the 63 supplements could be determined due to colour interference with the assay, as CD levels were determined spectrophotometrically. From the current analysis it seems that fewer supplements were in the early stages of rancidity during the 2012 study compared to the 2009 study.

**Fig. 5.**
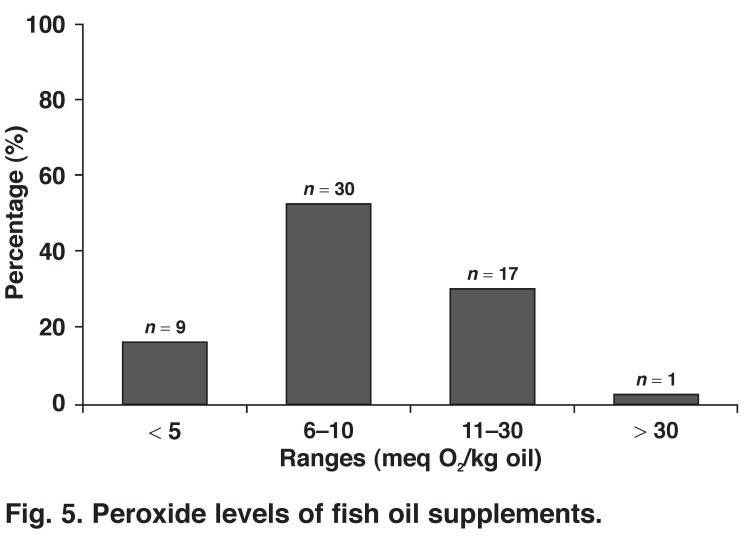
Peroxide levels of fish oil supplements.

Peroxide levels of < 5 meq O_2_/kg oil for edible oils are deemed acceptable, however, the majority (84%; *n* = 48) of the tested supplements contained peroxide levels higher than the acceptable range. Merely 16% (*n* = 9) of the fish oil supplements had peroxide levels within the proposed range (Fig. 5).

Peroxide levels of only 57 of the supplements could be determined due to colour interference with the assay, as peroxide levels were determined spectrophotometrically. Peroxide levels were not determined during the 2009 study.

**Fig. 6.**
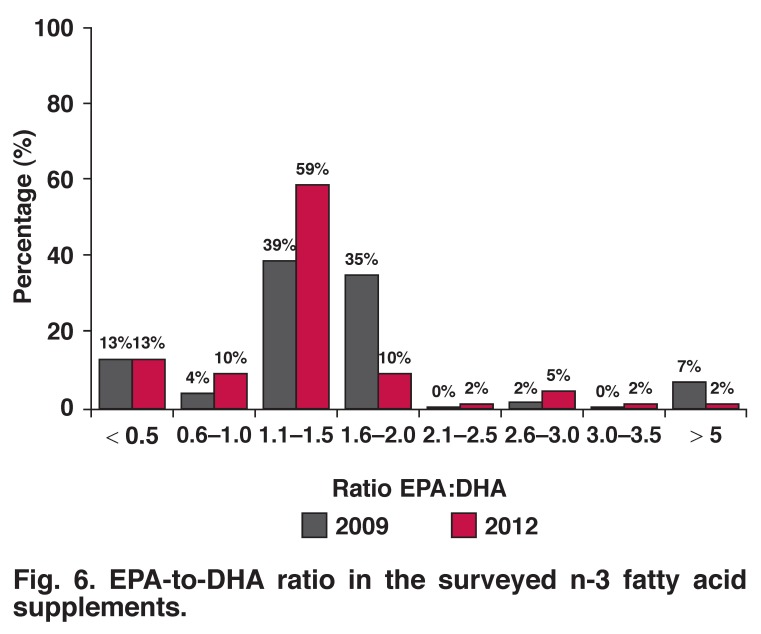
EPA-to-DHA ratio in the surveyed n-3 fatty acid supplements.

The majority (81%; *n* = 51) of the supplements under study had a 1.1–1.5:1 EPA-to-DHA ratio or less (Fig. 6). Only a few supplements had a 1.5–2.0:1 ratio compared to the results reported in the 2009 survey.

**Fig. 7.**
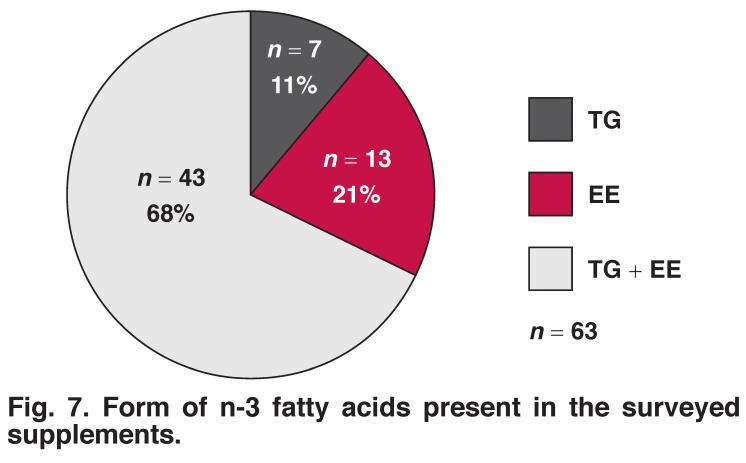
Form of n-3 fatty acids present in the surveyed supplements.

Fig. 7 shows that almost a third (32%; *n* = 20) of the analysed fish oil supplements available on the South African market contained EE or a combination of TG and EE. The form of the fatty acids was not determined during the 2009 study.

## Discussion

The choice of n-3 fatty acid supplements is expanding annually as more and more manufacturers enter the market. Some fish oil supplements sold in South Africa are encapsulated locally while all fish oil or fish oil concentrates are imported from different countries. Without proper local and international labelling legislation, in combination with strictly enforced manufacturing standards, the quality of fish oil supplements as well as the credibility of their labels remains questionable.

Other countries, for example the US and countries of the European Union (EU), have strict dietary supplement labelling legislation but in the US supplements are not subject to safety and efficacy testing requirements.^18^ The EU’s Food Supplements Directive^19^ requires that supplements are obliged to be demonstrated safe in terms of purity as well as the dosages recommended. Only supplements proved to be safe may be sold without a prescription. However, South Africa imports dietary supplements from many other countries that do not have to adhere to such strict regulations.

There was an improvement in the percentage (42% in 2012) of supplements that met the arbitrary range of 90–110% for EPA compared to the results of the previous survey (31% in 2009). With regard to DHA, there was a substantial increase in the percentage of supplements with DHA content > 110% (13% in 2009 vs 35% in 2012). This might be attributed to increased awareness of consumers regarding n-3 fatty acid intake and the emphasis on the importance of DHA for neural and brain development.

To increase the level of n-3 fatty acids in the diet with a fish oil supplement may be expensive for the average South African consumer. The current analysis shows that the majority of the surveyed supplements will cost the consumer R60 to R150 (R2.01 to R5.00/day) per person per month to attain the daily recommendation of 500 mg/day EPA + DHA, as stipulated by ISSFAL. For a family of four it will cost R240 to R600 per month, which makes fish oil supplementation unaffordable for the average South African household.

Rancidity is a concern, especially in polyunsaturated fatty acid oils such as fish oil. CDs were measured as an early indicator of rancidity. Almost half of the supplements tested were in the early stages of rancidity. Another concern is the peroxide levels of the majority of supplements, which were higher than the levels recommended by GOED^17^ (≤ 5 meq O_2_/kg oil). Peroxide levels are a measure of the oxidative rancidity of fats by determination of the lipid peroxides present.

Rancidity or oil degradation occurs when unsaturated fats are exposed to oxygen and become oxidised.^20^ Fish oil is prone to oxidation because of the presence of very long-chain polyunsaturated fatty acids compared to saturated fat or vegetable oils.^21^ Research^21^-^23^ has shown that dietary oxidised fatty acids pose an increased atherogenic risk. According to Turner *et al.*,^20^ the deleterious effects of oxidised fats on lipid and chylomicron metabolism, oxidative stress, inflammation, as well as vascular function contribute to the increased risk of atherosclerosis.

On average, fish oils contain 180 mg EPA and 120 mg DHA (ratio of 1.5:1) per 1 000 mg. In our previous analysis, the majority of EPA-to-DHA ratios of oils in the analysed supplements were 1.51–2.5:1, while in the current analysis more DHA was included in the supplements to supply oils with a 1.1–1.5:1 ratio. It is also evident that some of the surveyed supplements contained substantially more EPA and DHA per 1 000 mg oil compared with the 180 mg EPA + 120 mg DHA of standard fish oil. These so-called concentrated fish oils consist of EE which are the esterified products of fatty acids and ethanol. This chemical process is known as trans-esterification and involves the removal of the glycerol backbone of the TG in fish oil by substituting it with ethanol.

By using fractional distillation, which removes short-chain and saturated fatty acids from fish oil, it is now possible for manufacturers of supplements to allow for selective concentration of EPA and DHA to levels in excess of those found in natural fish oils. Esterification is also applied to deodorise the fish oil. These preparations are typically marketed as fish oil concentrates. Through the process of glycerolysis it is possible to convert EE back to TG. This process removes the ethanol molecule and re-esterifies the EPA and DHA fatty acids back to a glycerol backbone, resulting in the formation of re-esterified TG.

The digestion of EE is altered as a result of the absence of a glycerol backbone. Enzymes such as pancreatic lipase hydrolyse the fatty acids from the ethanol backbone in the small intestine. Since the fatty acid–ethanol bond of EE is much more resistant to hydrolysis by pancreatic lipase compared to the hydrolysis of TG in the natural form, this may explain the reduced bioavailability of EE. Because of the resistance to hydrolysis the digestion and absorption of EE is significantly lower compared to TG and phospholipids.

It is concerning that about a third of the surveyed n-3 fatty acid supplements analysed contained EE. Pregnant women are advised against the use of EE since the safety of EE during pregnancy has not been established. In this study, we analysed one supplement containing EE that specifically recommended it to be used during pregnancy. During the digestion of EE, they are converted back to TG and alcohol is released. Even though the quantity of ethanol released in a typical dose of fish oil via supplementation is small, at-risk groups such as alcoholics, pregnant women and young children should refrain from using n-3 fatty acid supplements that contain EE.

EE of fatty acids have shown toxicity *in vivo*^24^ and *in vitro*^25^ and are known to accumulate in major organs such as the heart, liver, pancreas and potentially the placenta.^26^ In a rat study,^27^ it was observed that efficient fatty acid EE digestion in the gastrointestinal tract may prevent toxicity, however, this was not confirmed in humans.

At present no manufacturers indicate the form (EE, TG and or rTG) in which n-3 fatty acids are present in capsules. The safety of daily intakes of EE and/or rTG via n-3 fatty acid supplements has not been confirmed in humans. EE and rTG are synthetic molecules and are not found naturally in food or the human body.

In some countries (not in South Africa) fatty acid EEs are used as prescription medication to reduce very high blood TG levels (≥ 5.65 mmol/l). However, these preparations provide pharmacological amounts of n-3 fatty acid EE and are used under strict supervision of a physician who should be well informed about the adverse effects the medicine might display. These drugs are also subjected to rigorous testing through three phases of clinical trials, as specified by the Federal Drug Administration. However, the same procedures are not applied to ensure quality, consistency and purity in dietary supplements.

By presenting n-3 fatty acid supplements in the form of EE or rTG, it enables manufacturers to increase the concentration of EPA and DHA in supplements. Our analysis has shown that manufacturers are increasing the DHA content of fish oil supplements to entertain the consumer’s demands for higher DHA intakes. The benefit of such higher DHA intakes needs further investigation.

A recent meta-analysis and systematic review^28^ reported that treatment with EPA or DHA showed diverse effects on high- and low-density lipoprotein cholesterol (HDL-C and LDL-C). Both treatments induced lower blood TG levels. However, DHA was more often associated with both elevated HDL-C levels and LDL-C levels. The increase in LDL-C is undesirable. On the other hand, clinical trials elsewhere^29^,^30^ indicated that fish oil supplementation is associated with increased LDL-C particle size or a shift in LDL-C particle distribution from small, dense particles to larger less dense LDL-C particles, which are known to be less atherogenic.

## Conclusion

Fish oil supplementation is a useful tool to increase the dietary intake of n-3 essential fatty acids. However, due to the absence of a food supplement regulatory structure in South Africa, there is concern about the safety, purity and quality of the fish oil supplements surveyed. This analysis has shown a small improvement in the accuracy of EPA content declared on supplement labels compared to the 2009 survey. It was also evident that a higher percentage of supplements contained DHA levels higher than declared.

Fish oil supplements remain expensive and are deemed to be unaffordable for the majority of South African consumers. A relatively large number of supplements were found to be in the early stages of rancidity. Of greater concern was the peroxide (rancidity) level of more than 80% of supplements, which were higher than the recommended content as specified by GOED.17 Dietary intakes of oxidised fatty acids may pose deleterious health effects.

Compared to the previous survey, it seems that manufacturers have increased the DHA-to-EPA ratio as well as the concentration of these two components. Some of the surveyed supplements had EPA and DHA contents much higher than the standard 180 mg EPA and 120 mg DHA per 1 000 mg fish oil. This can be attributed to the EE and/or rTG content of these preparations. The safety of EE and rTG has not been confirmed in humans.
